# Autoradiography of Intracerebral Tumours in the Chick Embryo Model: A Feasibility Study Using Different PET Tracers

**DOI:** 10.1007/s11307-025-01983-9

**Published:** 2025-01-21

**Authors:** Sandra Krause, Alexandru Florea, Chang-Hoon Choi, Wieland A. Worthoff, Alexander Heinzel, Saskia Fischer, Nicole Burda, Bernd Neumaier, N. Jon Shah, Philipp Lohmann, Felix M. Mottaghy, Karl-Josef Langen, Carina Stegmayr

**Affiliations:** 1 Institute of Neuroscience and Medicine (INM-4; INM-5; INM-11), Forschungszentrum Jülich, 52425 Jülich, Germany; 2 Department of Nuclear Medicine, RWTH Aachen University Hospital, Aachen, Germany; 3 Department of Neurology, RWTH Aachen University Hospital, Aachen, Germany; 4 JARA - BRAIN - Translational Medicine, Aachen, Germany; 5 Department for Nuclear Medicine, Martin Luther University Halle-Wittenberg, Halle (Saale), Germany; 6 Department of Radiology and Nuclear Medicine, Maastricht University Medical Center (MUMC+), Maastricht, The Netherlands

**Keywords:** Preclinical, Alternative, Chick embryo, Radiotracer, Glioma xenograft

## Abstract

**Purpose:**

In addition to rodent models, the chick embryo model has gained attention for radiotracer evaluation. Previous studies have investigated tumours on the chorioallantoic membrane (CAM), but its value for radiotracer imaging of intracerebral tumours has yet to be demonstrated.

**Procedures:**

Human U87 glioblastoma cells and U87-IDH1 mutant glioma cells were implanted into the brains of chick embryos at developmental day 5. After 12–14 days of tumour growth, blood–brain-barrier integrity was evaluated *in vivo* using MRI contrast enhancement or *ex vivo* with Evans blue dye. The tracers O-(2-[^18^F]fluoroethyl)-L-tyrosine ([^18^F]FET) (*n* = 5), 3,4-dihydroxy-6-[^18^F]-fluoro-L-phenylalanine ([^18^F]FDOPA) (*n* = 3), or [^68^Ga] labelled quinoline-based small molecule fibroblast activation protein inhibitor ([^68^Ga]FAPI-46) (*n* = 4) were injected intravenously if solid tumours were detected with MRI. For time-activity curves for [^18^F]FET, additional micro PET (µPET) was performed. The chick embryos were sacrificed 60 min post-injection, and cryosections of the tumour-bearing brains were produced and evaluated with autoradiography and immunohistochemistry.

**Results:**

Intracerebral tumours were produced with a 100% success rate in viable chick embryos at the experimental endpoint. However, 52% of chick embryos (*n* = 85) did not survive the procedure to embryonic development day 20. For the evaluated radiotracers, the tumour-to-brain ratios (TBR) derived from *ex vivo* autoradiography, as well as the tracer kinetics derived from µPET for intracerebral chick embryo tumours, were comparable to those previously reported in rodents and patients: the TBRmean for [^18^F]FET was 1.69 ± 0.54 (*n* = 5), and 3.8 for one hypermetabolic tumour and < 2.0 for two isometabolic tumors using [^18^F]FDOPA, with a TBRmean of 1.92 ± 1,11 (*n* = 3). The TBRmean of [^68^Ga]FAPI-46 for intracerebral chick embryo tumours was 19.13 ± 0.64 (*n* = 4). An intact blood-tumour barrier was observed in one U87-MG tumour (*n* = 5).

**Conclusions:**

Radiotracer imaging of intracerebral tumours in the chick embryo offers a fast model for the evaluation of radiotracer uptake, accumulation, and kinetics. Our results indicate a high comparability between intracerebral tumour imaging in chick embryos and xenograft rodent models or brain tumour patients.

**Supplementary Information:**

The online version contains supplementary material available at 10.1007/s11307-025-01983-9.

## Introduction

Rodent models have been widely used for *in vivo* oncology research because they can mimic complex metabolic pathways and organ-specific challenges, such as the blood–brain barrier (BBB) [1, 2]. In recent years, the chick embryo has been demonstrated to be a comparable alternative for xenograft glioma invasion [3–5]. Compared to rodent models, the utilisation of chick embryos is ethically preferential due to the phylogenetically lower stage of development. Legally, avian embryos are not considered under the European Directive on the protection of animals used for scientific purposes [6]. In Germany, recent evidence indicating nociception in later developmental stages of avian embryos [7–9] has led to a revision of animal protection laws. These revisions prohibit the termination of chick embryos starting from embryonic development day (EDD) 13 if gender determination has been performed [10]. However, chick embryo experiments that do not involve gender identification remain exempt from requiring an animal experimentation application. Furthermore, chick embryo research offers economic advantages, including lower costs and minimal animal husbandry requirements. Fertilised chick eggs are readily available, easy to manipulate, and allow for developmental synchronicity, enabling precise experimental control.

The chick chorioallantoic membrane (CAM), a highly vascularised, extra-embryonic membrane, is a widely-accepted research tool for tumour engraftments [11–15], allowing *in vivo* imaging, such as MRI and PET, for real-time monitoring of tumour growth and treatment effects [16–21]. While the CAM tumour model provides a convenient and rapid way to study tumour growth, the investigation of the interactions between tumours and the surrounding brain tissue *in vivo* requires orthotopic brain tumour implantation models. One approach is to implant cells into the neural tube of chick embryos in early development, allowing the growth and interaction of implanted cells in a more sophisticated *in vivo* model [22]. Another method uses a pressure injection of brain tumour cells into the midbrain at EDD 6, which also leads to the growth of intraventricular tumours [3]. However, both methods suffer from high failure rates or technically challenging injection procedures, and further optimisation of intracerebral tumour models in the chick embryo is required for widespread acceptance as an alternative *in vivo* model [16].

In this study, we explored a modified orthotopic chick embryo brain tumour implantation model by injecting tumour cells with a micro syringe into the mesencephalon. Our approach is based on established methods for rodent studies (using a binocular microscope inside the stereotactic frame holder) and does not require additional technical equipment. As proof of feasibility, tumoral tracer uptake and distribution, as well as the tracer kinetics of three clinically important radiotracers, were assessed in the chick embryo model and compared to results from mammalian models and brain tumour patients.

Firstly, we applied the amino acid radiotracer O-(2-[^18^F]fluoroethyl)-L-tyrosine ([^18^F]FET), which has proven to be a powerful tracer in brain cancer diagnostics, providing additional information on tumour metabolism, tumour extent, treatment effects and response assessment [23–26]. The extensive clinical and preclinical knowledge of [^18^F]FET uptake in brain tumours makes it an ideal radiotracer for evaluating the suitability of the chick embryo model in brain tumour imaging [24, 27–34]. In addition, [^68^Ga]-labelled quinoline-based small molecule fibroblast activation protein inhibitor ([^68^Ga]FAPI-46) was used to assess FAP expression. This protein is over-expressed in a number of neoplasms, particularly in epithelial cancers [35, 36] and gliomas [37, 38]. Although this tracer does not pass the intact BBB, it has been tested in intracerebral tumours with disrupted blood-tumour barriers, as FAP is implicated in tissue remodelling during embryogenesis and in reactive stromal cells [39]. This makes it particularly valuable for assessing potential limitations of the embryonic model, which does not replicate a mature biological system. Finally, another amino acid analogue with high relevance for the identification of metabolically active brain tumour tissues is 3,4-dihydroxy-6-[^18^F]-fluoro-L-phenylalanine ([^18^F]FDOPA). Similar to [^18^F]FET, [^18^F]FDOPA exhibits low uptake in healthy brain tissue but demonstrates additional uptake in the basal ganglia due to its role as a precursor in dopamine synthesis [40, 41].

Thus, this study aims to establish a more convenient intracerebral tumour model in the chick embryo model and provide the first examples of the use of this preclinical model for radiotracer evaluation using autoradiography, PET, CT, and MRI.

## Materials and Methods

### Treatment of Fertilised Eggs

Specific pathogen-free fertilised eggs for medical use (Valo BioMedia GmbH, Germany) were utilised for this study. Following arrival, the eggs were allowed to rest for at least 24 h at 14–18 °C. The eggs were then warmed at room temperature (22 ± 1 °C) for 12 h prior to incubation. Incubation was started on EDD 1 at 37.8 °C and a humidity of 54% (Favorit-Olymp 192, Heka, Germany). On EDD 5, a window was cut in the eggshell to access the embryo as described elsewhere (Supplemental Material) and subsequently sealed with adhesive tape. The eggs were incubated on their sides and not turned so that the albumen would not be spilt until the final experiments on EDD 18–20.

### Cell Culture

Gliomas with and without mutation of isocitrate dehydrogenase (IDH) were used here to help facilitate another ongoing study. U87 MG (ATCC® HTB-14™) and U87 IDH1^R132H^ (ATCC® HTB-14IG™) cells were cultured in Minimum Essential Medium Eagle containing 10% foetal calf serum, 1% L-glutamine, 1% MEM non-essential amino acids, and 1% penicillin/streptomycin, at 37 °C and 5% CO_2_.

The cells were resolved in the medium to a concentration of 5000/40 µl and cultured in a hanging drops culture under the lid of a petri dish for four days, resulting in cell pellets of approx. 50,000 cells, suitable for one injection. Just prior to implantation, one cell pellet was drawn under a binocular microscope into a neuro-syringe (Neuros™ 700/1700 Series, Hamilton, USA), connected to a stereotactic frame, with a volume ≤ 0.5 µl.

### Brain Tumour Implantation

**Fig. 1:**
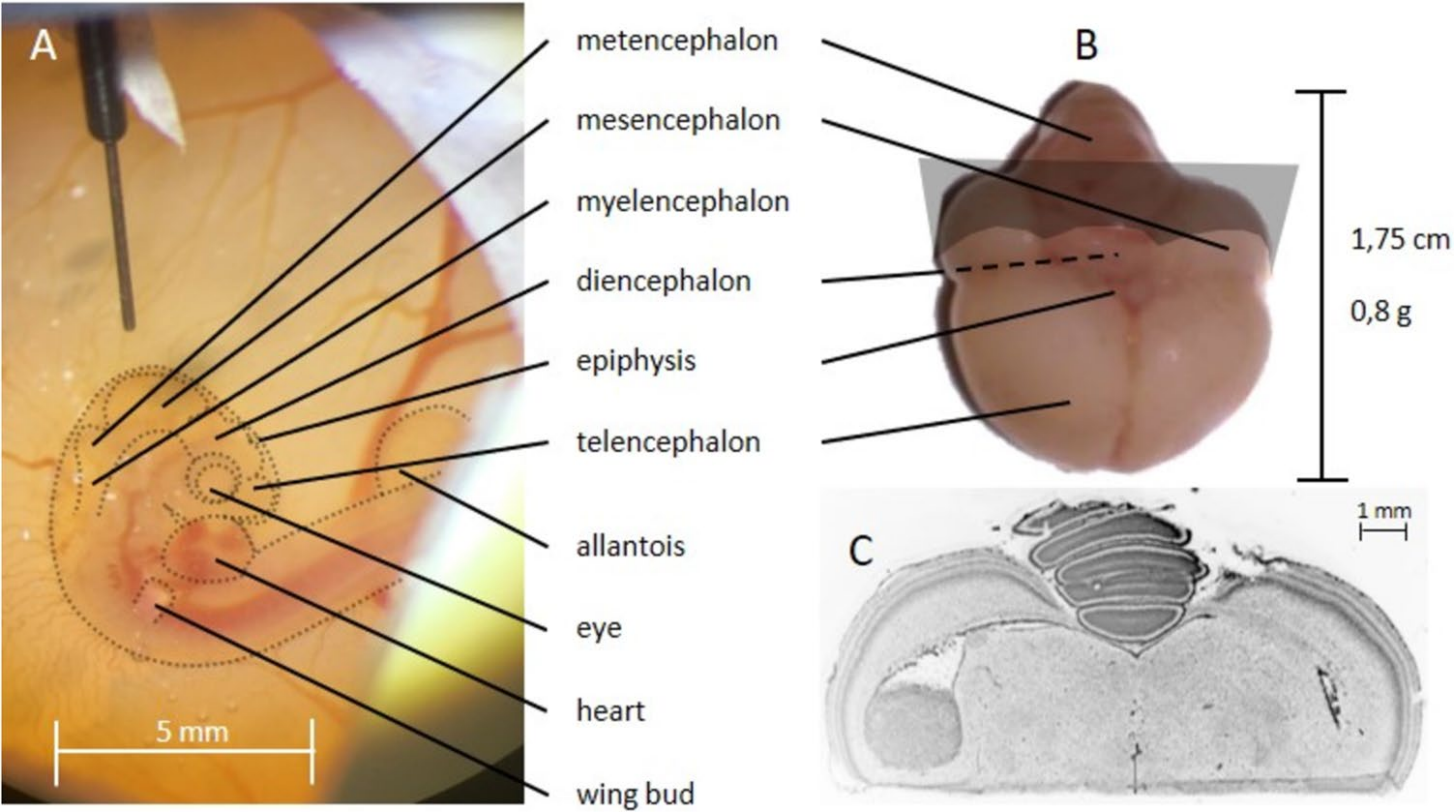
On EDD 5, brain tumour inoculation was performed by injecting tumour cells with a microsyringe into the mesencephalon (**A**). On EDD 19, brains were removed and cryosliced (**B**). Tumours were usually present on one or both optic tecti (grey plane in B), growing from the ventricle infiltratively into the brain tissue in a DAPI staining (**C**).

On EDD 5, the windowed egg was placed under the binocular microscope inside the stereotactic frame holding the neuro-syringe (Neuros™ 700/1700 Series, Hamilton, USA) to inject the tumour cells. With a thin, sharp glass capillary, the chorion was punctured and sliced next to the head of the embryo to gain access. Next, the spoon-end of a microspatula was positioned under the head to hold it in place while rupturing the amnion next to the head. For this, the tip of the glass capillary was used to press the amnion to the spatula and simultaneously pull it upwards the spatula until the amnion ruptured and the mesencephalon was exposed. With the head still lying on the spatula, the mesencephalon was punctured with the glass capillary. Depending on what was more comfortable, the head of the embryo either rested on the spatula or was released into its bedding before the neuro-syringe was lowered into the hole made into the mesencephalon (Fig. 1A). For this step, it is crucial that the amnion does not cover the hole, otherwise the tip of the syringe will be blocked and cells cannot be inserted into the mesencephalon. After injecting the cells into the brain, the syringe was pulled out, the extra-embryotic membranes (amnion and chorion) were replaced, and the window of the egg was covered with fresh tape. The membranes healed within the next 24–48 h, and a solid tumour grew inside the ventricles, infiltrating healthy brain tissue (Fig. 1B, C).

### Anaesthesia and Tracer Injection

For all imaging modalities, the egg was placed in a plexiglass cylinder, which was designed and constructed in-house to hold the anaesthetic. 15 MBq ± 5 MBq of O-(2-[^18^F]fluoroethyl)-L-tyrosine ([^18^F]-FET) (*n* = 3), 3,4-dihydroxy-6-[^18^F]-fluoro-L-phenylalanine ([^18^F]-FDOPA) (*n* = 3), or [^68^ Ga]-labelled quinoline-based small molecule fibroblast activation protein inhibitor ([^68^ Ga]-FAPI-46) (*n* = 4) was injected intravenously in 100 – 150 µL in 20 s, including 20 µL Evans Blue dye (2% in 0.9% NaCl solution) to identify BBB disruption, as described elsewhere [24]. The chick embryos were sacrificed 60 min post-injection. Imaging was performed under constant 2% isoflurane anaesthesia in oxygen, initialised with 5% isoflurane in oxygen for 5–10 min, to mitigate embryo movement. For the MR scans, 120 µL isoflurane was pipetted onto tissue paper and placed inside the plexiglass cylinder holding the MRI radiofrequency coil and the egg to reach approximately 2% isoflurane in air; the cylinder was then sealed immediately. To maintain anaesthesia at 2% isoflurane during the PET and CT scans, the plexiglass cylinder was connected to a small animal isoflurane vaporiser. Whenever possible, the temperature was maintained with an infrared lamp.

To facilitate good access, the eggs were opened as wide as the spread of the CAM would allow before the commencement of the injections. All substances were injected intravenously into a middle-sized vein of the CAM, which are the light red vessels with blood flow from smaller to bigger vessels. Substances were either injected via a catheter (tip of a 30 G cannula connected to a PE tube) placed in the CAM vessel or directly using a 1 ml syringe with a 32 G or 33 G cannula. The rate of successful intravenous injection was increased to > 95% through the utilisation of the following tools: more accurate injection was facilitated by employing a stereomicroscope (5–8 magnification) and a syringe. Prior to retracting the cannula, a few drops of policresulen (360 mg/g) were administered to the injection site to prevent heavy bleeding if a bigger cannula and vessel were used for injection. However, using 32 G or 33 G cannulas for veins not bigger than the cannula itself negates the need to use policresulen, as pinching the vessel for a few seconds stops potential bleeding.

### MR Imaging

MRI was used to verify brain tumour growth before tracer experiments and to evaluate BBB integrity after the injection of a paramagnetic contrast agent. Embryos with adequate-sized tumours in T1 and T2w images were chosen for PET and autoradiography the next day. All MR experiments were conducted at a 7 T clinical whole-body scanner (Siemens Healthineers, Erlangen, Germany) on EDD 18–19. In order to maximise the MR sensitivity and coverage, we designed, constructed, and used a low-pass birdcage radio-frequency coil and interface specifically for egg measurements. Further details are given in Supplemental Material and Figure S1A.

### PET/CT Imaging and Autoradiography

Dynamic PET scans were performed with an INVEON scanner (Siemens, Erlangen, Germany) [42] or a Triumph II CT scanner (Northridge Tri-Modality Imaging, USA) for 65 min ([^18^F]FET). If the chick embryos were injected under a stereomicroscope, emission scans started 3 min after the injection, followed by 10 min of transmission scan (INVEON) or a low dose CT (Triumph II) for attenuation correction. If the chick embryos were injected via a venous catheter (INVEON only), transmission scans were performed first, followed by emission scans with simultaneous tracer injection. Details on PET reconstruction are given in Supplemental Material. An image of the PET scan setup is shown in Figure S1B.

After the scan, the chick embryos were checked for vital signs (*i.e.*, visual assessment of pulse in CAM arteries under the stereomicroscope) before further processing of the tissue and data. For autoradiography, organs were taken out, frozen in isopentane at −50 °C, and cryo-cut in 20 µm slices. Every tenth slice and freshly prepared 20 μm ^18^F or ^68^ Ga standards with known activity for a calibration curve were exposed to an imaging plate (Fuji Imaging Plate, Raytest) overnight, scanned (Fuji BAS Reader 5000, Raytest), and evaluated for tracer uptake with a pixel size of 25 μm (AIDA Version 4.50, Raytest).

### Immunohistochemistry

The brains were removed from the chick embryos, frozen in isopentane at −50 °C, and cryo-cut in 20 µm coronal slices. Slices were fixed with paraformaldehyde and subsequently stained for glial fibrillary acid protein (*i.e.,* anti-GFAP, DAKO Z0334), Ki-67 (ab16667, Abcam), human nuclei (MAB1281, Merck Millipore) and nuclei (*i.e.*, DAPI) using the standard protocols for fixed cryo-slices. Quantification of proliferative cells was performed on overview images of Ki-67 stained brain slices using ImageJ (National Institute of Health, Bethesda, MD, United States). Evans blue dye extravasation was acquired by fluorescence and pictured using an Aida Image Analyzer (AIDA Version 4.50; Raytest-Fuji).

### Statistics

All statistical calculations were performed using GraphPad Prism 10.0.2 (GraphPad Software, Inc., La Jolla, CA, United States). Descriptive statistics are provided as mean and standard deviation (SD). A paired t-test and Pearson correlation (r) were chosen for methodology comparison. A p-value of less than 0.05 was considered to indicate significant statistical differences in all tests.

## Results

**Fig. 2:**
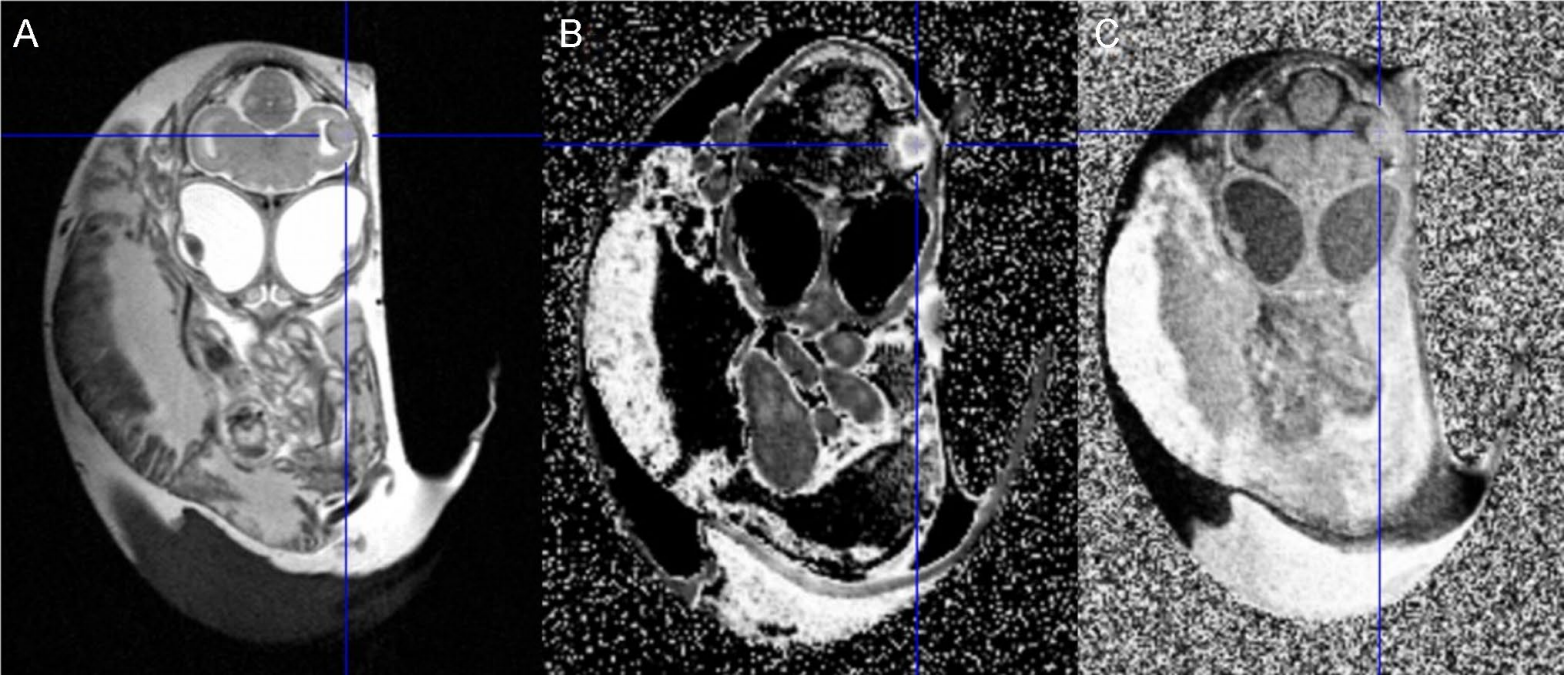
MR images of an EDD 19 chick embryo. A U87-MG tumour can be seen in the optic tectum (crosshair), presented in T2 (**A**) and T1 with (**B**) and without (**C**) contrast agent. Contrast agent enhancement is most profound in the tumour-rim region.

**Fig. 3:**
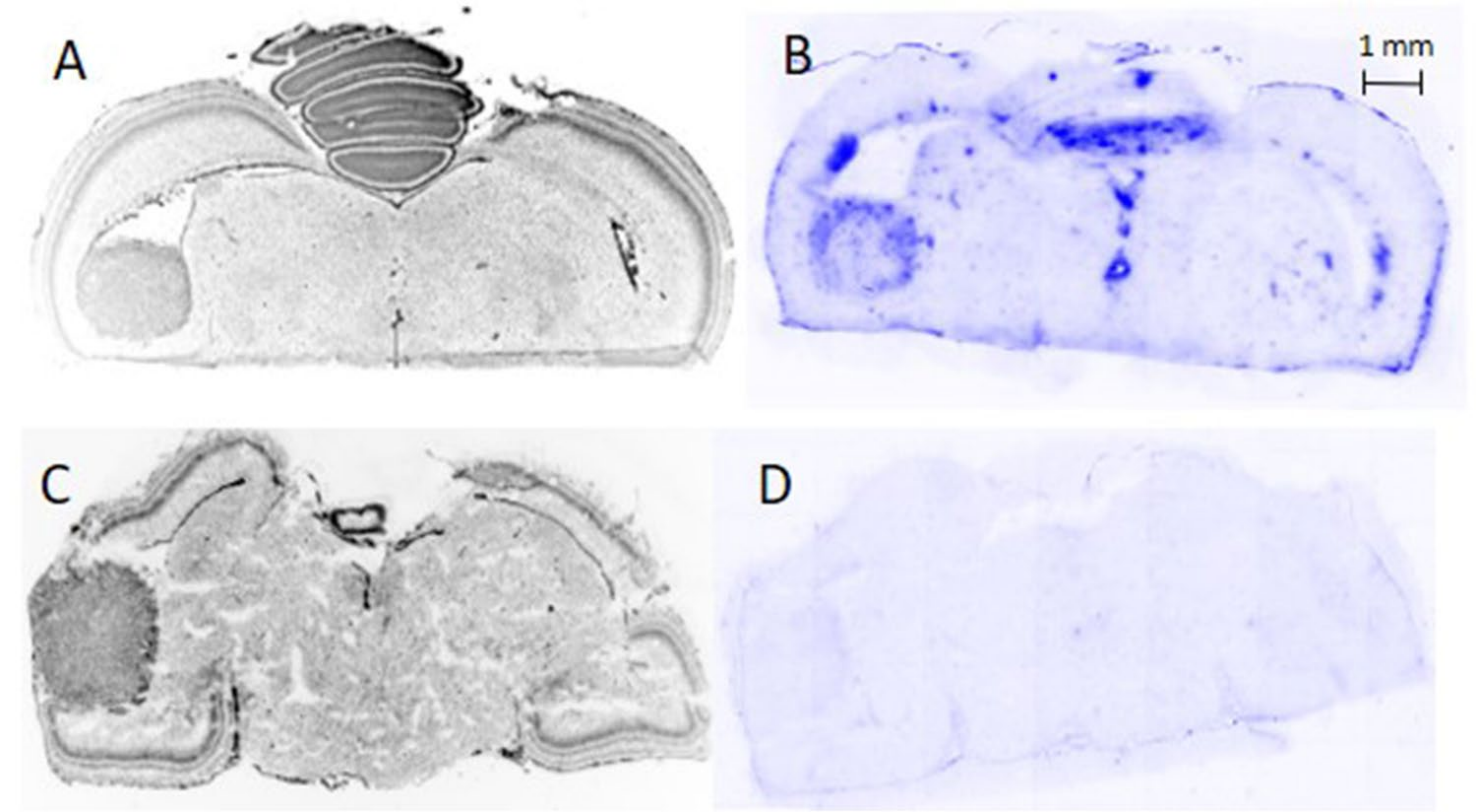
Two representative EDD 19 chick embryo brains with U87-MG tumours showing a disrupted (**A,B**) and an intact (**C,D**) blood-tumour-barrier. **A,C:** 4’,6-diamidino-2-phenylindole (DAPI) staining shows densely packed tumour cells in the tectum opticum. **B,D:** Consecutive slices of A and C, respectively, with (**B**) and without (**D**) Evans blue dye extravasation in the tumour region, thus indicating a disrupted (**B**) and an intact (**D**) blood-tumour-barrier.

Intracerebral tumours were produced with a success rate of 100% in viable chick embryos at the experimental endpoint. However, 52% of embryos did not survive the procedure to EDD20 (*n* = 85 in general). Without intervention, the mortality rate was 13.3%. Sample sizes of each experiment are shown in Table 1. Tumour cells were reliably found within one or both optic tecti growing from the ventricle into the brain tissue infiltratively (Fig. 1C). The BBB integrity was tested either *in ovo* with MRI after injection of paramagnetic contrast agent (Fig. 2) or *ex ovo* using Evans blue extravasation (Fig. 3). In cases with BBB disruption, Evans blue dye extravasation could be observed in the tumour region, whereas Evans blue dye uptake was not seen in tumours with an intact blood-tumour barrier. An intact blood-tumour barrier was observed in one U87-MG intracerebral tumour (*n* = 5), which was used in the [^18^F]FET study. No intact blood-tumour barriers were found in the tested U87-IDH1^R132H^ tumours (*n* = 7).

**Table 1:** Sample sizes of each experiment, as well as related mortality rates (where applicable). Developmental failure refers to the loss of chick embryo viability during embryonic development. Windowing on EDD5 serves as the baseline without intervention

Subexperiment	Sample size n [-]	Developmental failure n [-]	Mortality rate [%]
Tumour cell implantation in the chick embryo brain	85	44	51.76
Windowing on EDD5, based on survival until EDD10	226	30	13.3
[^18^F]FET autoradiography	5		
[^18^F]FDOPA autoradiography	3		
[^68^Ga]FAPI autoradiography	4		
[^18^F]FET dynamic PET	5

**Fig. 4:**
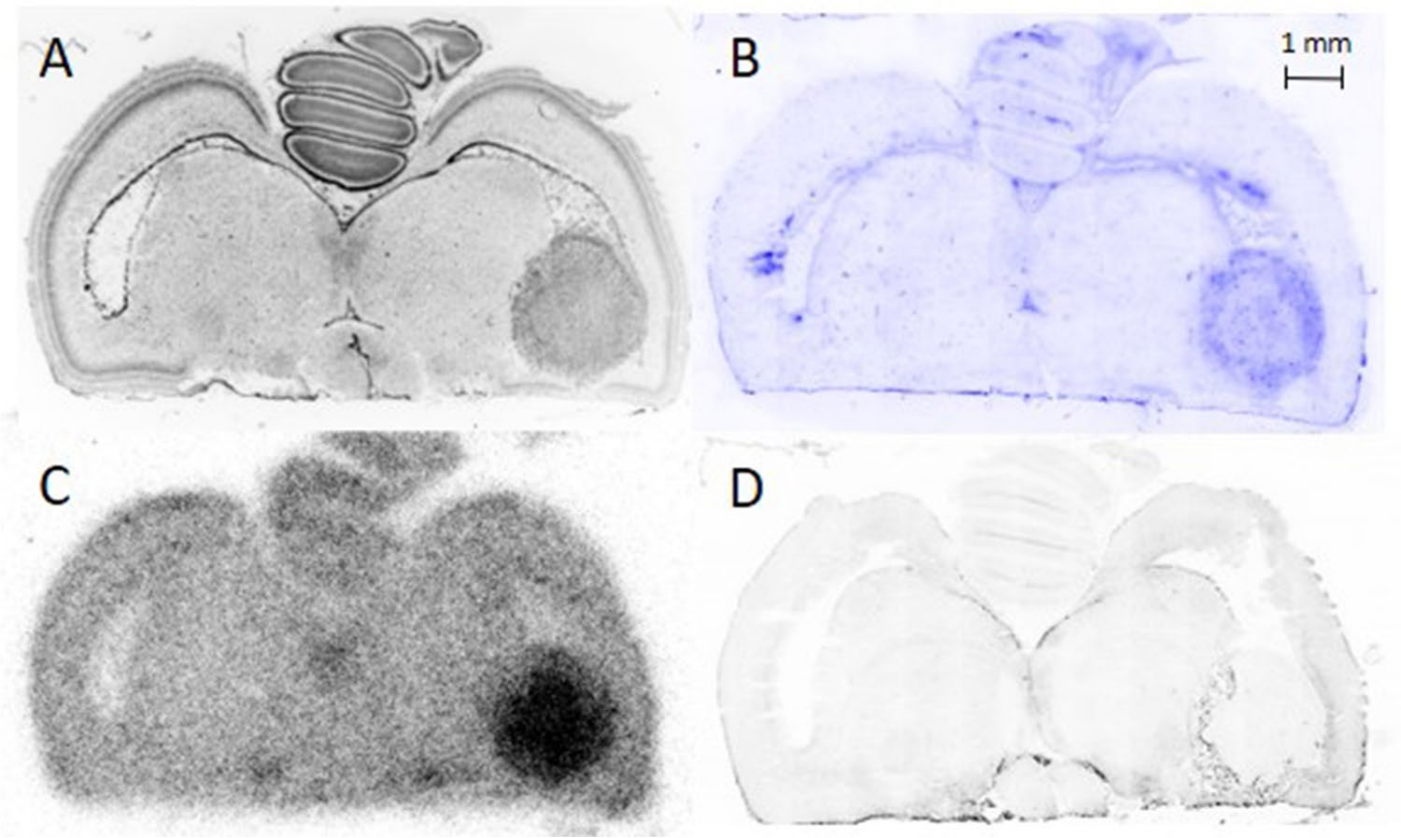
Consecutive 20 µm slices of an EDD 19 chick embryo brain with a U87-MG tumour using [^18^F]FET. 4’,6-diamidino-2-phenylindole (DAPI) staining shows densely packed tumour cells in the ventricle and adjacent brain regions forming a solid tumour (**A**). The blood–brain barrier is disrupted within the tumour, as shown by Evans blue dye extravasation, especially in the tumour rim region (**B**). The corresponding [^18^F]FET autoradiography shows a profound tracer accumulation in the tumour that is independent of the degree of blood–brain-barrier disruption (**C**). Astrocyte staining using an anti-GFAP antibody (Z0334, Dako) reveals astrocyte activation in the vicinity of the tumour (**D**).

The mean tumour-to-brain ratio (TBRmean) of [^18^F]FET uptake in intracerebral U87 MG tumours in chick embryos was 1.69 ± 0.54, as determined by *ex vivo* autoradiography which showed high correlation efficiency to PET-derived values (Figure S2). A representative overview of the histological DAPI staining with densely packed tumour cells, BBB disruption within the tumour via Evans blue dye extravasation, [^18^F]FET accumulation within the tumour, as well as astrocyte activation in the vicinity of the tumour via immunostaining using anti-GFAP antibody, can be seen in Fig. 4. The TBRs derived from *ex vivo* autoradiography were comparable to those previously reported in rodents and patients (Table 2). A detailed comparison of derived values for [^18^F]FET compared to rodent xenografts and brain tumour patients is given in the Supplementary Table S1.

**Table 2:** Tumour-to-brain ratios (TBRmean) of the evaluated tracers in different species, in mean with standard deviation

	Chick Embryo	Rodents	Human
[^18^F]FET	1.69 ± 0.54	2.15 ± 0.37 [29]	2.02 ± 0.54 [28]
[^18^F]FDOPA	1.92 ± 1.11	2.41 to up to 3.36 [60]	1.76 ± 0.60 [61]
[^68^Ga]FAPI-46	19.13 ± 0.64		19.95 ± 13.22 [38]

Dynamic [^18^F]FET PET imaging using small animal PET allowed the assessment of the time-activity curves (TAC) of tracer accumulation in different organs (*i.e.*, heart, liver, kidney, and brain). (Figure S3). The plateau of the TAC of [^18^F]FET was reached about 20 min after intravenous injection for all investigated organs. [^18^F]FET showed the highest tracer uptake in the heart (SUV_heart_ = 3.70 ± 0.35), followed by the liver (SUV_liver_ = 3.51 ± 0.41 and kidneys (SUV_kidney_ = 3.26 ± 0.43), indicating [^18^F]FET clearance through hepatic/pancreatic and urinary pathway. The SUV within the brain was moderate, with an average of 1.09 ± 0.09. A representative image is shown in Figure S4.

**Fig. 5:**
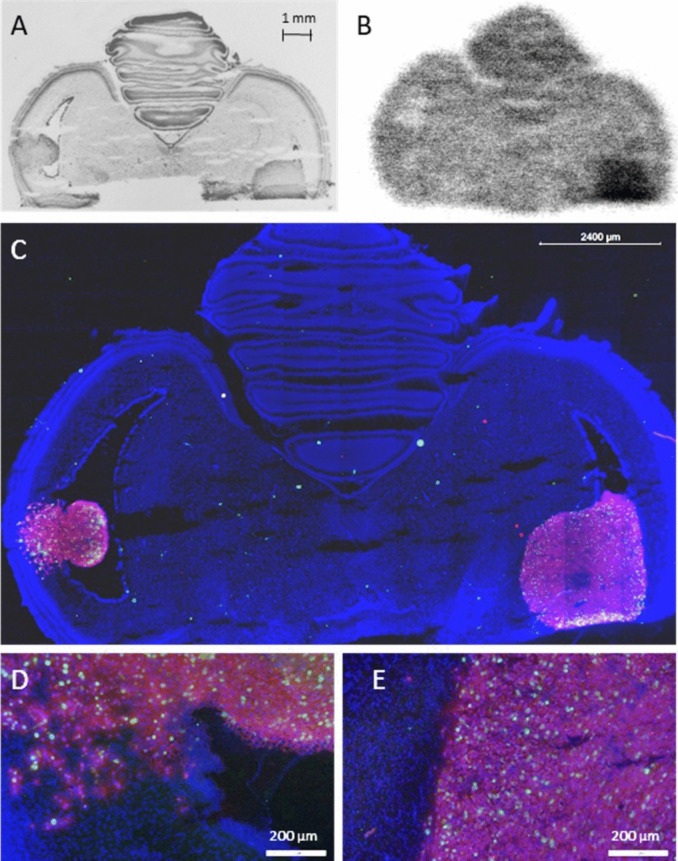
Consecutive 20 µm slices of an EDD 19 chick brain with a U87-IDH1 R132H tumour. 4’,6-diamidino-2-phenylindole (DAPI) staining shows densely packed tumour cells in both ventricles and adjacent brain regions that form two solid tumours (**A**). *Ex vivo* autoradiography with [^18^F]FDOPA shows a profound tracer accumulation in the right tumour, while the left tumour visualises isometabolism (**B**). Immunostaining with a human nuclei marker (MAB1281, Merck Millipore) demonstrates the human origin of the tumours in red, whereas proliferating cells are visualised in green with the proliferation marker Ki-67 (ab16667, Abcam). All cell nuclei can be seen in blue (**C**). Detailed images of the border region of both the isometabolic left tumour (**D**) and hypermetabolic right tumour (**E**) are shown in higher magnification.

An interesting example of *ex vivo* autoradiography using [^18^F]FDOPA in a chick embryo bearing two U87-IDH1^R132H^ tumours in the right and left ventricle of each tectum is shown in Fig. 5. The right-sided tumour showed high tracer uptake (TBR, 3.8), while the left showed isometabolism (TBR, 1.1). A chick-genetic origin of one of the tumours was excluded by immunostaining using a human nuclei marker (Fig. 5C, red). Both tumours showed high proliferative indices (Fig. 5C, green), with significant differences in proliferation density in the infiltration zone: 37% proliferative cells for the isometabolic tumour but 62% Ki-67 positive cells in the FDOPA positive tumour. Detailed images visualise the infiltrative growth pattern of the isometabolic tumour into the brain of the chick embryo (Fig. 5D), while the hypermetabolic tumour shows a solid border of the tumour and brain (Fig. 5E). For intracerebral U87-MG (*n* = 2), one chick brain tumour also showed isometabolism (TBR, 1.1), whereas the tumour of the other subject exhibited higher uptake (TBR, 1.7).

**Fig. 6:**
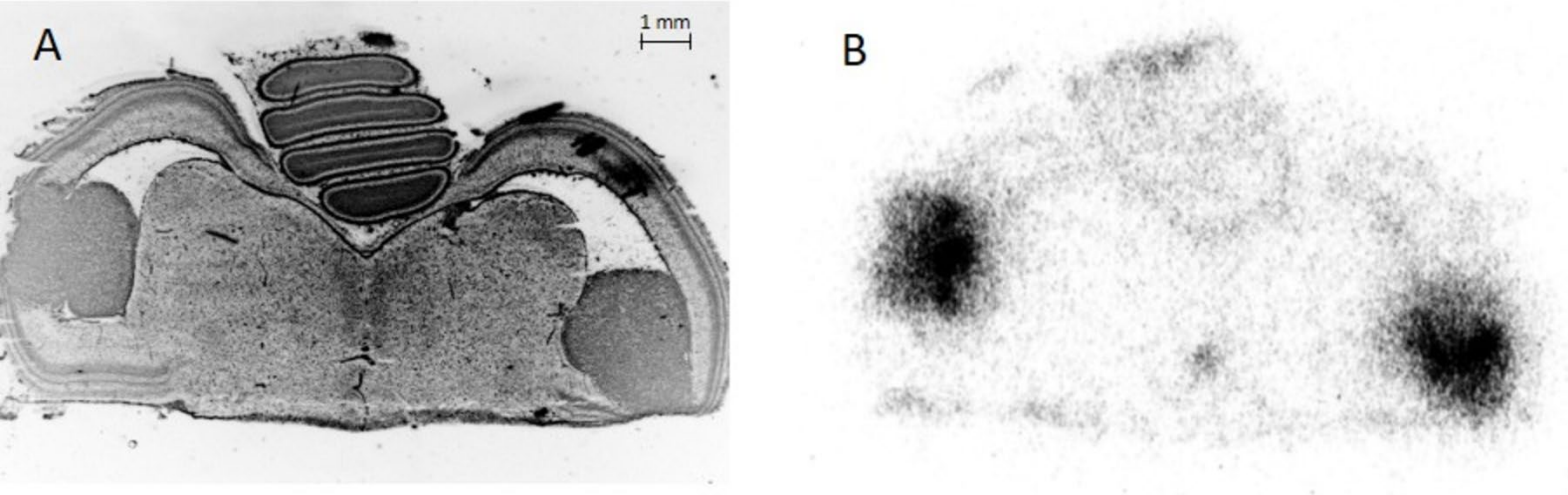
[^68^Ga]FAPI-46 of a chick embryo on EDD 19. (**A**) 4’,6-diamidino-2-phenylindole (DAPI) image of U87 IDH1^R132H^ intracerebral tumour showing densely packed solid tumour cells in the ventricles (**B**) autoradiography of consecutive slice of A, showing profound tracer accumulation in the tumours.

An example of the distribution of [^68^Ga]FAPI-46 in a chick embryo brain in a U87 IDH1^R132H^ intracerebral tumour at EDD 19 is shown in Fig. 6. The TBR of [^68^Ga]FAPI-46 uptake in U87-IDH1^R132H^ tumours with disrupted blood-tumour barriers (*n* = 4) was 19.13 ± 0.64.

## Discussion

The presented chick embryo model for intracerebral tumours yielded solid brain tumours inside the ventricles of the optic tectum of the chick embryo, infiltrating healthy brain tissue. The growth and interaction of implanted cells with the surrounding developing brain tissue was similar to established rodent brain tumour models.

Autoradiographic experiments with the amino acid tracer [^18^F]FET showed low uptake in the normal brain and TBR values similar to those reported in the literature for both humans [28] and rats [24, 43]. The occurrence of one U87-MG tumour with an intact blood-tumour barrier did not influence the tracer uptake due to the ability of [^18^F]FET to pass an intact BBB. In addition, the time-activity curves (Figure S3) determined by the small animal PET yielded similar results compared with rodents and humans, i.e., decreasing [^18^F]FET uptake after an early peak in most organs, while the brain showed SUVs in the range of 1 in rats, humans, and chick embryos. The whole-body distribution and [^18^F]FET clearance from the organs of the chick embryo were also comparable to known elimination pathways in rodents and humans. In mice, urinary and pancreatic excretion has been shown [44], while human dynamic whole-body scans showed urinary excretion with high [^18^F]FET accumulations in the urinary bladder and kidneys [45]. Also, a noticeable amount of [^18^F]FET activity was found in the human myocardium [45]. In chick embryos, the highest [^18^F]FET accumulation was found in the heart, followed by high liver and kidney uptakes shortly after distribution. This might indicate a [^18^F]FET clearance through both hepatic and kidney / urinary excretion within the chick embryo on EDD 18–19. However, pancreatic excretion cannot be ruled out due to the anatomically close proximity of the liver and pancreas [46].

Experiments with [^18^F]FDOPA yielded brain tumours with different uptake behaviour in the same cell lines, which is a rather unusual finding (Fig. 5). This variability suggests that brain tumour cells in the chick embryo model may display differing levels of differentiation, providing an opportunity to further investigate the mechanisms underlying tracer uptake. Low uptake of FET or FDOPA also occurs in up to 30% of brain tumour patients [47], resembling the isometabolic uptake in the chick embryo (Fig. 5). The proliferative index of the chick embryo isometabolic tumour was lower than the chick embryo hypermetabolic tumour, which is in line with the finding that patients with isometabolic brain tumours tend to have a more favourable prognosis [47, 48].

Finally, [^68^Ga]FAPI-46 uptake within the chick embryo at the later EDDs was similar to the uptake reported in patients [38]. At this embryonic stage, FAPI uptake within the embryonic brain was as low as in the mouse model [37]. Tissue remodelling during embryogenesis and reactive stromal cells showed high uptake in the jaw muscle and spine (data not shown). However, this did not lead to increased FAPI uptake in the brain, suggesting that the embryonic origin did not limit the model’s utility in brain tumour studies.

Thus, all three radiotracers showed similar results compared with those in rodents and humans, indicating that the chick embryo model is an excellent preclinical model for tracer evaluation in intracerebral tumours.

Compared with previously published techniques for brain tumour implantation [3, 4, 22, 49, 50], our method is based on established methods for rodent studies, is technically less challenging, and has comparably moderate chick embryo mortality rates. A recent study proposed a pneumatic pico-pump for glioblastoma cell injection in embryos at EDD 6 with viable brain tumours in 25 – 75% of the experiments and evaluation of tumour growth up to EDD16 [5]. Our approach yielded a similar rate of embryo viability (48%) and allowed the evaluation of intracerebral tumour growth and infiltration up to EDD20, which is advantageous for therapy monitoring studies.

The formation of a BBB in chick embryos has been described in several studies [51–55]. Impairment of the BBB within the area of the brain tumour could be detected in later EDDs, similar to that observed with established rat models with the same tumour cell line [24]. As demonstrated in Fig. 3, however, some intracerebral tumours in the chick embryo brains did not show signs of BBB disruption, which is similar to non-enhancing gliomas in humans. Since an intact BBB is rarely found in rodent brain tumour models, this observation indicates the potential of the chick embryo as a model for non-enhancing gliomas.

Some limitations that restrict the applicability of the chick embryo model need to be addressed. First, the implantation site in the developing mesencephalon of the chick embryo brain varies from rodent models as the striatum is not developed at the time of implantation. However, the presence of intermediate filament protein GFAP expression in the vicinity of the solid brain tumour indicates reactive astrocytosis in response to tumour growth, which is comparable to that observed in mammals [56–59]. Thus, the tumour microenvironment seems to be comparable to that seen in rodent xenografts. However, due to the limited spatial resolution of the small animal PET, a clear delineation of tumours was not possible. Therefore, the tumour-bearing chick embryo brain was investigated using *ex vivo* autoradiography on EDD 18–19. Nevertheless, a significant correlation between mean SUV and TBR values derived using PET and autoradiography could be demonstrated (Figure S2). Another limitation is the restricted timeframe for experimental investigations. The fast development of chick embryos (20 days) excludes the investigation of slow-growing tumours or, in the case of drug testing, the use of drugs with longer administration times. Ethical considerations present a significant constraint in chick embryo research, requiring attention to ensure ethically correct treatment of these organisms during their development based on recent research indicating nociception starting from EDD13 [7–9]. While the utilisation of chick embryos might be ethically preferable to working with mature animals, an ethical dilemma persists, and researchers must continue to apply the 3R principles to minimise potential pain or harm. A balance between pursuing scientific knowledge and an ethical imperative has to be found. As an example, all embryos sacrificed in this study were anaesthetised beforehand as part of a refinement strategy. Additionally, the research planning incorporated measures to minimise the number of embryos used. Based on patient data and observations in rodent xenograft models, medium-sized brain tumours of the type developed in this study are not associated with pain. Therefore, no pain, suffering or harm during the developmental stages with possible nociception was anticipated. However, continuous reassessment and refinement of protocols based on the newest findings is imperative to uphold the highest standards of animal welfare. In addition, a notable limitation of the model arises from the inherent differences between avian and mammalian immunobiology, particularly in cases where host receptor-binding is required for specific radiotracers or when antibodies are used for immunohistochemical target validation. Nonetheless, if the target binding is restricted to the derived tumour itself, it does not pose a limitation to the evaluation of tracer developments or neuroimaging, as the xenografted human-originated tumour still retains its human physiological properties. In the context of host immunological processes, the extrapolation of findings from chick embryo studies to potential applications in mammalian systems must be undertaken with caution, and conclusions should be drawn carefully. Consequently, the translational relevance of immunological results obtained in chick embryos may be limited and should be interpreted carefully.

## Conclusions

Radiotracer imaging of intracerebral tumours in the chick embryo offers a fast model for the evaluation of radiotracer uptake, accumulation, and kinetics. Our results indicate a high comparability of chick embryo intracerebral tumour imaging to xenograft rodent models or brain tumour patients. The results of multimodal imaging, as well as the tumour growth and micro-environment, indicate an excellent *in vivo* alternative to the standard rodent model in many preclinical aspects of neuro-oncology and non-invasive medical imaging. Furthermore, the occurrence of isometabolic tumours, as well as intact blood-tumour barriers, offer new options to study the role of blood–brain-barrier permeability for the uptake of radiotracers.

## Supplementary Information

Below is the link to the electronic supplementary material.

Supplementary file1 (DOCX 1746 KB)

## Data Availability

All research data and computer codes are available from the corresponding author upon request.

## References

[1] Pardridge WM (2005) The blood-brain barrier: bottleneck in brain drug development. Neurotherapeutics 2(1):3–14. 10.1602/neurorx.2.1.3 10.1602/neurorx.2.1.3 PMC539316 15717053

[2] Day C-P, Merlino G, van Dyke T (2015) Preclinical mouse cancer models: a maze of opportunities and challenges. Cell 163(1):39–53. 10.1016/j.cell.2015.08.068 26406370 10.1016/j.cell.2015.08.068 PMC4583714

[3] Cretu A, Fotos JS, Little BW, Galileo DS (2005) Human and rat glioma growth, invasion, and vascularization in a novel chick embryo brain tumor model. Clin Exp Metastasis 22(3):225–236. 10.1007/s10585-005-7889-x 16158250 10.1007/s10585-005-7889-x

[4] Pace KR, Dutt R, Galileo DS (2019) Exosomal L1CAM stimulates glioblastoma cell motility, proliferation, and invasiveness. Int J Mol Sci 20(16):3982. 10.3390/ijms20163982 31426278 10.3390/ijms20163982 PMC6720723

[5] Pastorino NG, Tomatsu S, Lin A et al (2023) Using the chick embryo brain as a model for in vivo and ex vivo analyses of human glioblastoma cell behavior. J Vis Exp (195):e65199. 10.3791/65199 10.3791/65199 37306427

[6] European Union (2010) Directive 2010/63/EU of the European Parliament and of the Council of 22 September 2010 on the protection of animals used for scientific purposes. OJL 276:33–79. ELI: http://data.europa.eu/eli/dir/2010/63/oj

[7] Kollmansperger S, Anders M, Werner J et al (2023) Nociception in chicken embryos, part II: Embryonal development of electroencephalic neuronal activity in ovo as a prerequisite for nociception. Animals (Basel) 13(18):2839. 10.3390/ani13182839 37760239 10.3390/ani13182839 PMC10525651

[8] Süß SC, Werner J, Saller AM et al (2023) Nociception in chicken embryos, part III: Analysis of movements before and after application of a noxious stimulus. Animals (Basel) 13(18):2859. 10.3390/ani13182859 37760259 10.3390/ani13182859 PMC10525827

[9] Weiss L, Saller AM, Werner J et al (2023) Nociception in chicken embryos, part I: Analysis of cardiovascular responses to a mechanical noxious stimulus. Animals (Basel) 13(17):2710. 10.3390/ani13172710 37684974 10.3390/ani13172710 PMC10486618

[10] Tierschutzgesetz (TierSchG) - German Animal Welfare Act of 24 July 1972 in Federal Law Gazette I (BGBl. I p. 1277), last amended on 16 May 2022 (BGBl. I p.742)

[11] Strojnik T, Kavalar R, Barone TA, Plunkett RJ (2010) Experimental model and immunohistochemical comparison of U87 human glioblastoma cell xenografts on the chicken chorioallantoic membrane and in rat brains. Anticancer Res 30:4851–60 21187462

[12] Miebach L, Berner J, Bekeschus S (2022) In ovo model in cancer research and tumor immunology. Front Immunol 13:1006064. 10.3389/fimmu.2022.1006064 36248802 10.3389/fimmu.2022.1006064 PMC9556724

[13] Harper K, Yatsyna A, Charbonneau M et al (2021) The chicken chorioallantoic membrane tumor assay as a relevant in vivo model to study the impact of hypoxia on tumor progression and metastasis. Cancers (Basel) 13(5):1093. 10.3390/cancers13051093 33806378 10.3390/cancers13051093 PMC7961795

[14] Smith LM, Greenwood HE, Tyrrell WE et al (2023) The chicken chorioallantoic membrane as a low-cost, high-throughput model for cancer imaging. Npj Imaging 1(1):1. 10.1038/s44303-023-00001-3 38239706 10.1038/s44303-023-00001-3 PMC7615542

[15] Benčurová K, Tran L, Friske J et al (2024) An in vivo tumour organoid model based on the chick embryonic chorioallantoic membrane mimics key characteristics of the patient tissue: a proof-of-concept study. EJNMMI Res 14(1):86. 10.1186/s13550-024-01151-0 39331331 10.1186/s13550-024-01151-0 PMC11436503

[16] Winter G, Koch ABF, Löffler J et al (2020) Multi-modal PET and MR imaging in the hen’s egg test-chorioallantoic membrane (HET-CAM) model for initial in vivo testing of target-specific radioligands. Cancers (Basel) 12(5):1248. 10.3390/cancers12051248 32429233 10.3390/cancers12051248 PMC7281765

[17] Chen L, Wang S, Feng Y et al (2021) Utilisation of chick embryo chorioallantoic membrane as a model platform for imaging-navigated biomedical research. Cells 10(2):463. 10.3390/cells10020463 33671534 10.3390/cells10020463 PMC7926796

[18] Zuo Z, Syrovets T, Wu Y et al (2017) The CAM cancer xenograft as a model for initial evaluation of MR labelled compounds. Sci Rep 7:46690. 10.1038/srep46690 28466861 10.1038/srep46690 PMC5413881

[19] Würbach L, Heidrich A, Opfermann T, Gebhardt P, Saluz HP (2012) Insights into bone metabolism of avian embryos in ovo via 3D and 4D 18F-fluoride positron emission tomography. Mol Imaging Biol 14(6):688–698. 10.1007/s11307-012-0550-6 22422564 10.1007/s11307-012-0550-6

[20] Warnock G, Turtoi A, Blomme A et al (2013) In vivo PET/CT in a human glioblastoma chicken chorioallantoic membrane model: a new tool for oncology and radiotracer development. J Nucl Med 54(10):1782–1788. 10.2967/jnumed.112.117150 23970367 10.2967/jnumed.112.117150

[21] Schulze J, Schöne L, Ayoub AM et al (2023) Modern photodynamic glioblastoma therapy using curcumin- or parietin-loaded lipid nanoparticles in a CAM model study. ACS Appl Bio Mater 6(12):5502–5514. 10.1021/acsabm.3c00695 38016693 10.1021/acsabm.3c00695 PMC10732153

[22] Boulland J-L, Halasi G, Kasumacic N, Glover JC (2010) Xenotransplantation of human stem cells into the chicken embryo. J Vis Exp (41):2071. 10.3791/2071 10.3791/2071 PMC3144657 20644515

[23] Galldiks N, Langen K-J (2015) Applications of PET imaging of neurological tumors with radiolabeled amino acids. Q J Nucl Med Mol Imaging 59:70–82 25517079

[24] Stegmayr C, Oliveira D, Niemietz N et al (2017) Influence of bevacizumab on blood-brain barrier permeability and O-(2–18F-Fluoroethyl)-l-tyrosine uptake in rat gliomas. J Nucl Med 58(5):700–705. 10.2967/jnumed.116.187047 28153956 10.2967/jnumed.116.187047

[25] Langen K-J, Stoffels G, Filss C et al (2017) Imaging of amino acid transport in brain tumours: Positron emission tomography with O-(2–18Ffluoroethyl)-L-tyrosine (FET). Methods 130:124–134. 10.1016/j.ymeth.2017.05.019 28552264 10.1016/j.ymeth.2017.05.019

[26] Singnurkar A, Poon R, Detsky J (2023) 18F-FET-PET imaging in high-grade gliomas and brain metastases: a systematic review and meta-analysis. J Neurooncol 161(1):1–12. 10.1007/s11060-022-04201-6 36502457 10.1007/s11060-022-04201-6

[27] Stegmayr C, Willuweit A, Lohmann P, Langen K-J (2019) O-(2–18F-Fluoroethyl)-L-tyrosine (FET) in neurooncology: a review of experimental results. Curr Radiopharm 12(3):201–210. 10.2174/1874471012666190111111046 30636621 10.2174/1874471012666190111111046

[28] Stegmayr C, Stoffels G, Kops ER et al (2019) Influence of dexamethasone on O-(2–18F-Fluoroethyl)-L-tyrosine uptake in the human brain and quantification of tumor uptake. Mol Imaging Biol 21(1):168–174. 10.1007/s11307-018-1221-z 29845426 10.1007/s11307-018-1221-z

[29] Stegmayr C, Bandelow U, Oliveira D et al (2017) Influence of blood-brain barrier permeability on O-(2–18F-fluoroethyl)-L-tyrosine uptake in rat gliomas. Eur J Nucl Med Mol Imaging 44(3):408–416. 10.1007/s00259-016-3508-0 27613541 10.1007/s00259-016-3508-0

[30] Tang G, Wang M, Tang X, Luo L, Gan M (2003) Pharmacokinetics and radiation dosimetry estimation of O-(2–18Ffluoroethyl)-L-tyrosine as oncologic PET tracer. Appl Radiat Isot 58(2):219–225. 10.1016/s0969-8043(02)00311-1 12573321 10.1016/s0969-8043(02)00311-1

[31] Richard MA, Fouquet JP, Lebel R, Lepage M (2017) Determination of an optimal pharmacokinetic model of 18F-FET for quantitative applications in rat brain tumors. J Nucl Med 58(8):1278–1284. 10.2967/jnumed.116.180612 28765227 10.2967/jnumed.116.180612

[32] Wang H-E, Wu S-Y, Chang C-W et al (2005) Evaluation of F-18-labeled amino acid derivatives and 18FFDG as PET probes in a brain tumor-bearing animal model. Nucl Med Biol 32(4):367–375. 10.1016/j.nucmedbio.2005.01.005 15878506 10.1016/j.nucmedbio.2005.01.005

[33] Maurer GD, Brucker DP, Stoffels G et al (2020) 18F-FET PET imaging in differentiating glioma progression from treatment-related changes: a single-center experience. J Nucl Med 61(4):505–511. 10.2967/jnumed.119.234757 31519802 10.2967/jnumed.119.234757

[34] Lohmann P, Herzog H, Rota Kops E et al (2015) Dual-time-point O-(2-(18)Ffluoroethyl)-L-tyrosine PET for grading of cerebral gliomas. Eur Radiol 25(10):3017–3024. 10.1007/s00330-015-3691-6 25813014 10.1007/s00330-015-3691-6

[35] Rosenkrans ZT, Massey CF, Bernau K et al (2022) 68 GaGa-FAPI-46 PET for non-invasive detection of pulmonary fibrosis disease activity. Eur J Nucl Med Mol Imaging 49(11):3705–3716. 10.1007/s00259-022-05814-9 35556159 10.1007/s00259-022-05814-9 PMC9553066

[36] Chandekar KR, Prashanth A, Vinjamuri S, Kumar R (2023) FAPI PET/CT imaging-an updated review. Diagnostics (Basel) 13(12):2018. 10.3390/diagnostics13122018 37370912 10.3390/diagnostics13122018 PMC10297281

[37] Röhrich M, Loktev A, Wefers AK et al (2019) IDH-wildtype glioblastomas and grade III/IV IDH-mutant gliomas show elevated tracer uptake in fibroblast activation protein-specific PET/CT. Eur J Nucl Med Mol Imaging 46(12):2569–2580. 10.1007/s00259-019-04444-y 31388723 10.1007/s00259-019-04444-y

[38] Yao Y, Tan X, Yin W et al (2022) Performance of 18 F-FAPI PET/CT in assessing glioblastoma before radiotherapy: a pilot study. BMC Med Imaging 22(1):226. 10.1186/s12880-022-00952-w 36566187 10.1186/s12880-022-00952-w PMC9789562

[39] Jacob M, Chang L, Puré E (2012) Fibroblast activation protein in remodeling tissues. Curr Mol Med 12(10):1220–1243. 10.2174/156652412803833607 22834826 10.2174/156652412803833607

[40] Roach JR, Plaha P, McGowan DR, Higgins GS (2022) The role of 18Ffluorodopa positron emission tomography in grading of gliomas. J Neurooncol 160(3):577–589. 10.1007/s11060-022-04177-3 36434486 10.1007/s11060-022-04177-3 PMC9758109

[41] Walker MD, Dinelle K, Kornelsen R et al (2013) In-vivo measurement of LDOPA uptake, dopamine reserve and turnover in the rat brain using 18FFDOPA PET. J Cereb Blood Flow Metab 33(1):59–66. 10.1038/jcbfm.2012.120 22929441 10.1038/jcbfm.2012.120 PMC3597374

[42] Bao Q, Newport D, Chen M, Stout DB, Chatziioannou AF (2009) Performance evaluation of the inveon dedicated PET preclinical tomograph based on the NEMA NU-4 standards. J Nucl Med 50(3):401–408. 10.2967/jnumed.108.056374 19223424 10.2967/jnumed.108.056374 PMC2803022

[43] Stegmayr C (2016) Effect of pharmacological interventions on reproducibility of O-(2-[18F]fluoroethyl)-L-tyrosine (FET) uptake kinetics in rat glioma models. Doctoral dissertation, RWTH Aachen University. Available at: https://www.biologie.rwth-aachen.de/cms/biologie/Forschung/Publikationen/Publikationen/~fmtva/Details/?file=568281&lidx=1

[44] Heiss P, Mayer S, Herz M, Wester H-J, Schwaiger M, Senekowitsch-Schmidtke R (1999) Investigation of transport mechanism and uptake kinetics of O-(2-[18F]Fluoroethyl)-L-tyrosine in vitro and in vivo. J Nucl Med 40(8):1368–1373 10450690

[45] Pauleit D, Floeth F, Herzog H et al (2003) Whole-body distribution and dosimetry of O-(2–18Ffluoroethyl)-L-tyrosine. Eur J Nucl Med Mol Imaging 30(4):519–524. 10.1007/s00259-003-1118-0 12589478 10.1007/s00259-003-1118-0

[46] Bellairs R, Osmond M (2014) The atlas of chick development, 3rd edn. Academic Press, Oxford. 10.1016/C2010-0-65149-2

[47] Galldiks N, Unterrainer M, Judov N et al (2019) Photopenic defects on O-(2–18F-fluoroethyl)-L-tyrosine PET: clinical relevance in glioma patients. Neuro Oncol 21(10):1331–1338. 10.1093/neuonc/noz083 31077276 10.1093/neuonc/noz083 PMC6784268

[48] Galldiks N, Verger A, Zaragori T et al (2019) Comment on “Hypometabolic gliomas on FET-PET-is there an inverted U-curve for survival?” Neuro Oncol 21(12):1612–1613. 10.1093/neuonc/noz173 31504819 10.1093/neuonc/noz173 PMC6917398

[49] Boulland J-L, Leung DSY, Thuen M et al (2012) Evaluation of intracellular labeling with micron-sized particles of iron oxide (MPIOs) as a general tool for in vitro and in vivo tracking of human stem and progenitor cells. Cell Transplant 21(8):1743–1759. 10.3727/096368911X627598 22490338 10.3727/096368911X627598

[50] Cage TA, Louie JD, Liu SR, Alvarez-Buylla A, Gupta N, Hyer J (2012) Distinct patterns of human medulloblastoma dissemination in the developing chick embryo nervous system. Clin Exp Metastasis 29(4):371–380. 10.1007/s10585-012-9456-6 22322278 10.1007/s10585-012-9456-6

[51] Wakai S, Hirokawa N (1978) Development of the blood-brain barrier to horseradish peroxidase in the chick embryo. Cell Tissue Res 195(2):195–203. 10.1007/BF00236719 737715 10.1007/BF00236719

[52] Janzer RC, Raff MC (1987) Astrocytes induce blood-brain barrier properties in endothelial cells. Lettto Nat 325:253–7 10.1038/325253a0 3543687

[53] Möller W, Kummer W (2003) The blood-brain barrier of the chick glycogen body (corpus gelatinosum) and its functional implications. Cell Tissue Res 313(1):71–80. 10.1007/s00441-003-0742-0 12768407 10.1007/s00441-003-0742-0

[54] Parvas M, Parada C, Bueno D (2008) A blood-CSF barrier function controls embryonic CSF protein composition and homeostasis during early CNS development. Dev Biol 321(1):51–63. 10.1016/j.ydbio.2008.05.552 18632096 10.1016/j.ydbio.2008.05.552

[55] Parvas M, Bueno D (2010) The embryonic blood-CSF barrier has molecular elements to control E-CSF osmolarity during early CNS development. J Neurosci Res 88(6):1205–1212. 10.1002/jnr.22293 19937806 10.1002/jnr.22293

[56] Lee J, Borboa AK, Baird A, Eliceiri BP (2011) Non-invasive quantification of brain tumor-induced astrogliosis. BMC Neurosci 12:9. 10.1186/1471-2202-12-9 21247490 10.1186/1471-2202-12-9 PMC3033849

[57] Piroth MD, Prasath J, Willuweit A et al (2013) Uptake of O-(2–18Ffluoroethyl)-L-tyrosine in reactive astrocytosis in the vicinity of cerebral gliomas. Nucl Med Biol 40(6):795–800. 10.1016/j.nucmedbio.2013.05.001 23769262 10.1016/j.nucmedbio.2013.05.001

[58] Sofroniew MV, Vinters HV (2010) Astrocytes: biology and pathology. Acta Neuropathol 119(1):7–35. 10.1007/s00401-009-0619-8 20012068 10.1007/s00401-009-0619-8 PMC2799634

[59] Chekhonin VP, Baklaushev VP, Yusubalieva GM, Pavlov KA, Ukhova OV, Gurina OI (2007) Modeling and immunohistochemical analysis of C6 glioma in vivo. Bull Exp Biol Med 143(4):501–509. 10.1007/s10517-007-0167-y 18214311 10.1007/s10517-007-0167-y

[60] Clément A, Zaragori T, Filosa R et al (2022) Multi-tracer and multiparametric PET imaging to detect the IDH mutation in glioma: a preclinical translational in vitro, in vivo, and ex vivo study. Cancer Imaging 22(1):16. 10.1186/s40644-022-00454-6 35303961 10.1186/s40644-022-00454-6 PMC8932106

[61] Cicone F, Filss CP, Minniti G et al (2015) Volumetric assessment of recurrent or progressive gliomas: comparison between F-DOPA PET and perfusion-weighted MRI. Eur J Nucl Med Mol Imaging 42(6):905–915. 10.1007/s00259-015-3018-5 25750084 10.1007/s00259-015-3018-5

