# Supplementary material for: Autoradiography of Intracerebral Tumours in the Chick Embryo Model: A Feasibility Study Using Different PET Tracers
Source: Mol Imaging Biol. 2025 Jan 21;27(2):151–62. doi: 10.1007/s11307-025-01983-9 (PMC12062108; doi:10.1007/s11307-025-01983-9)
Supplement: Supplementary file 1 — Supplementary file1 (DOCX 1746 KB) [file 11307_2025_1983_MOESM1_ESM.docx]

**Electronic Supplementary Material**

**Autoradiography of intracerebral tumours in the chick embryo model: A feasibility study using different PET tracers**

**Journal: Molecular Imaging and Biology**

Sandra Krause,^1^ Alexandru Florea,^2,6^ Chang-Hoon Choi,^1^ Wieland A. Worthoff,^1^ Alexander Heinzel,^1,5^ Saskia Fischer,^1^ Bernd Neumaier,^1^ N. Jon Shah,^1,3,4^ Felix M. Mottaghy,^2,4,6^ Philipp Lohmann,^1,2^ Karl-Josef Langen,^1,2^ Carina Stegmayr,^1,2^

1. Inst. of Neuroscience and Medicine (INM-4; INM-5; INM-11), Forschungszentrum Jülich, Jülich, Germany
2. Dept. of Nuclear Medicine, RWTH Aachen University Hospital, Aachen, Germany
3. Dept. of Neurology, RWTH Aachen University Hospital, Aachen, Germany
4. JARA - BRAIN - Translational Medicine, Aachen, Germany
5. Dept. for Nuclear Medicine, Martin Luther University Halle-Wittenberg, Halle (Saale), Germany
6. Dept. of Radiology and Nuclear Medicine, Maastricht University Medical Center (MUMC+), Maastricht, The Netherlands

**Corresponding author:**

Sandra Krause

Institute of Neuroscience and Medicine

Forschungszentrum Jülich

Jülich, Germany, 52428

Tel: +49-(2461)-61-6941

Email: [san.krause@fz-juelich.de](mailto:san.krause@fz-juelich.de)

**Materials and Methods**

Windowing

On EDD 5, a window was cut in the eggshell to access the embryo as follows: A hole was drilled (Mikro 8050, Dremel, USA) into the air cell, and 2 ml of albumen was removed with a syringe at an angle of 45° or more, so as not to damage the yolk. By gently rotating the egg, the air bubble was moved from the blunt end into the albumen. This was controlled by candling the egg. With the egg laying on its side, a window (1 cm × 2 cm) was made above the new air cell using a cutting wheel. Both the hole and the window were sealed with adhesive tape. The eggs were incubated on their sides and not turned so that the albumen would not be spilled until the final experiments on EDD 18-20.

MR imaging

The inner diameter and length of the coil are 45 mm and 60 mm, respectively. The coil and egg were fitted perfectly inside a plastic cylindrical case (74 mm inner diameter × 160 mm length), as shown in Figure S2A. The egg was anesthetised in the case to avoid any movement. To acquire contrast-enhanced T1 weighted MR imaging, 30 µL of 0.5 mmol/ml gadopentetic acid was injected intravenously into a CAM vessel. Two sets of standard MR sequences (MP2RAGE for T1 weighted with/without the contrast agent and turbo spin echo (TSE) for T2 weighted) were employed, and their parameters are shown below.

The protocol of 3D MR2RAGE was repetition time (TR): 4310 ms, echo time (TE) = 3 ms, number of averages = 2, resolution = 0.2 mm^3^ isotropic, and acquisition time = 22:13 minutes. The 2D TSE protocol was TR = 6340 ms, TE = 52 ms, number of averages = 4, slice thickness = 0.7 mm, in-plane resolution = 0.1 × 0.1 mm^2^, acquisition time = 13:25 minutes.

PET data analysis

Image analysis was performed in summed images (18-50 min post injection for ^18^F tracer, 15-30 min post injection for ^68^Ga tracer) using PMOD (Version 4.205, PMOD Technologies Ltd.). Volumes of interest (VOIs) were placed by manually outlining the respective tissues of interest, *i.e*., heart, liver, kidney, and brain.

The mean tumor-to-brain ratio (TBR) was calculated by dividing the tracer uptake in tumor tissue by the uptake in normal brain tissue in the contralateral hemisphere. The mean standardized uptake value (SUVmean) was expressed by dividing the radioactivity in the tissue by the injected radioactivity per gram of body weight. The whole egg was chosen as the reference body weight (47-51 g). Time-activity curves for tracer uptake in respective tissues were determined.

PET data acquisition

Dynamic PET data acquisition was performed for 65 min ([^18^F]FET). OSEM3D/MAP was applied to reconstruct an image voxel size of 0.7764 × 0.7764 × 0.796 mm^3^ (matrix size: 128 × 128 × 159) for the INVEON, and a voxel size of 0.25 × 0.25 × 0.597 mm^3^ (matrix size: 240 × 240 × 192) for the Triumph II. Images were corrected for decay, scatter, attenuation, and dead time.

**Results**

**Table S1. Relevant [^18^F]FET parameters in different species**

|  | **SUV_brain_**  **[-]** | **SUV_tumor_ [-]** | **TBR_mean_ [-]** | **TBR_max_ [-]** | **Sample size [-]** | **Method of determination** | **Citation** |
| --- | --- | --- | --- | --- | --- | --- | --- |
| **Human** | 1.06 ± 0.16 | 2.06 | 2.02 ± 0.54 | 2.67 ± 1.07 | 80 | PET | (1) |
| **Rat** | 0.67 ± 0.14 | 1.42 ± 0.42 | 2.15 ± 0.37 |  | 5 | AR | (2) |
| **Chick embryo** | 0.80 ± 0.31 | 1.25 ± 0.35 | 1.69 ± 0.54 | 1.92 ± 0.5 | 5 | AR | here |
|  | As body weight reference, the whole egg was chosen. | |  |  |  |  |  |

**Figures:**

**
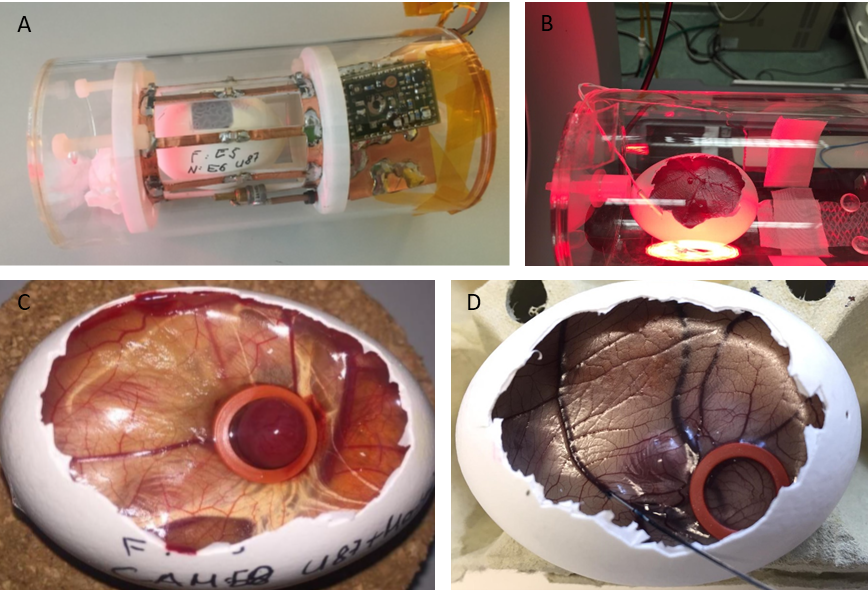
**

**Figure S1.** Overview of the methods. A plexiglass cylinder holds both the MR coil and the anesthetic gas during the MR scan, as a constant flow of isoflurane was not possible in the setup of a clinical scanner **(A)**. The same cylinder was also used for PET scans in a preclinical setup with a constant flow of isoflurane. A heating lamp was used to maintain approx. 37°C **(B)**.


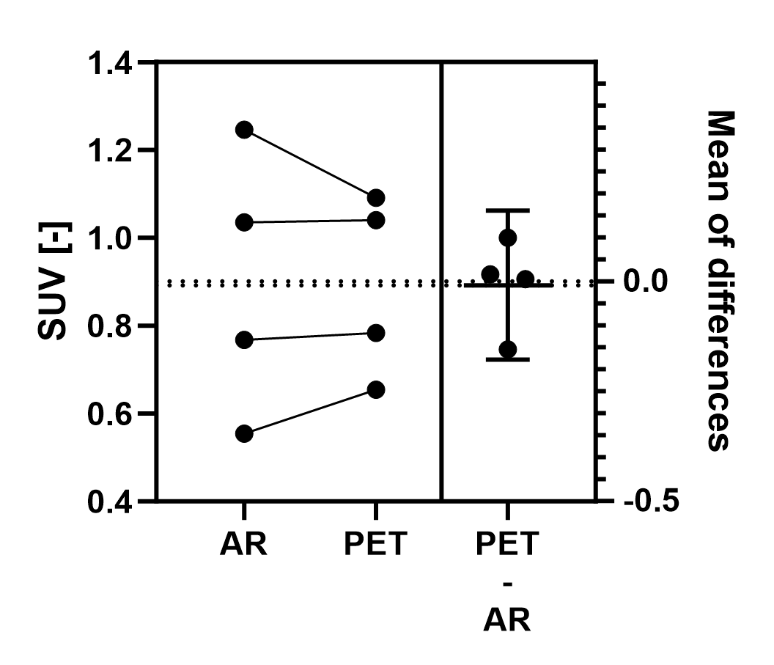


**Figure S2:** Correlation efficiency of PET and *ex vivo* autoradiography (AR) derived standardized uptake values of 4 subjects, showing significantly effective pairing (p = 0.8840, two-tailed; correlation coefficient r = 0.9811 with p = 0.0094).

**
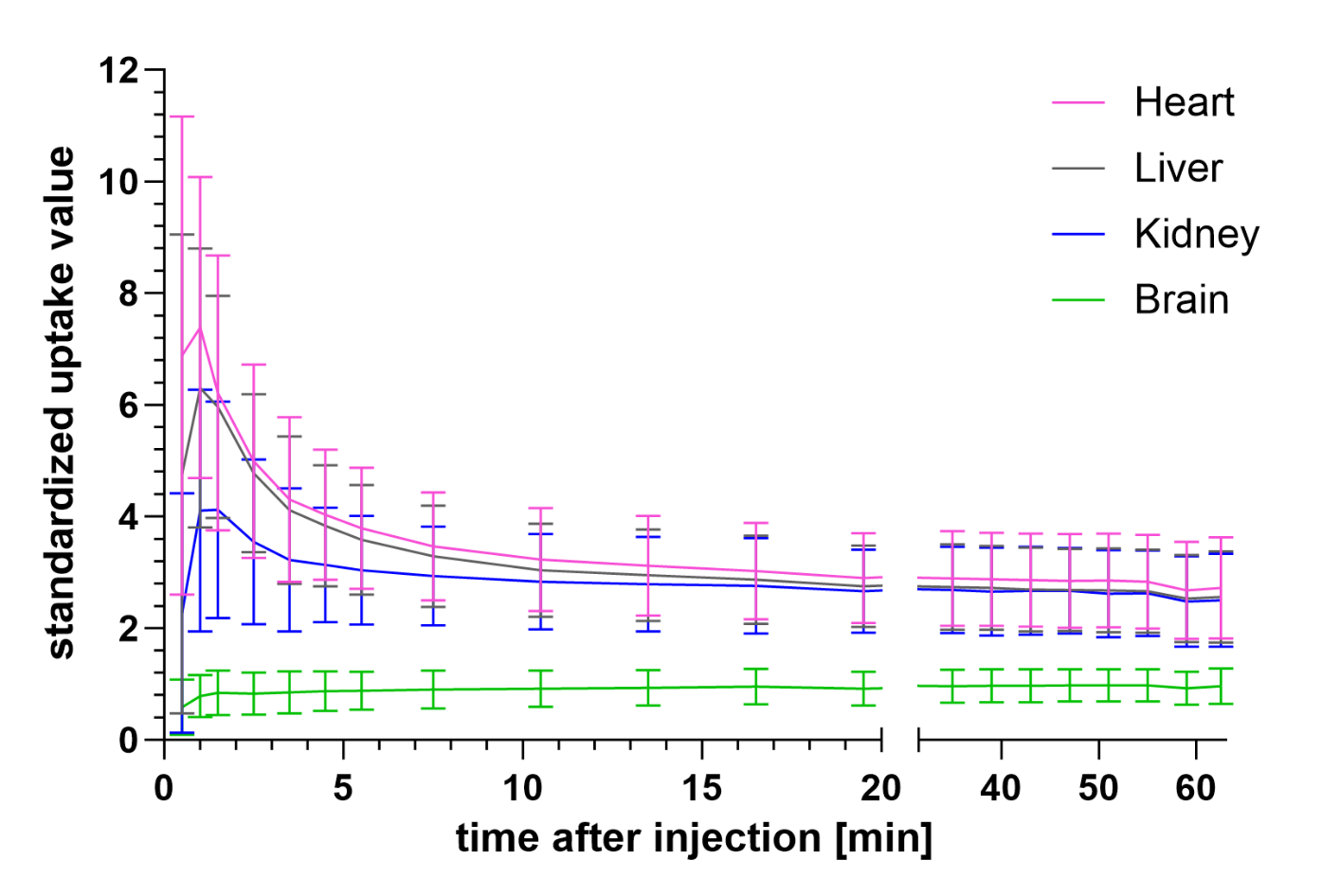
**

**Figure S3.** Averaged (n = 5) time-activity curves of O-(2-[^18^F]-fluoroethyl)-L-tyrosine uptake in the chick embryo model in the heart, liver, kidney, and brain. High mean uptake values in the liver and kidney could be observed within the first minutes of injection. The organ uptake reached equilibrium after 20 minutes.


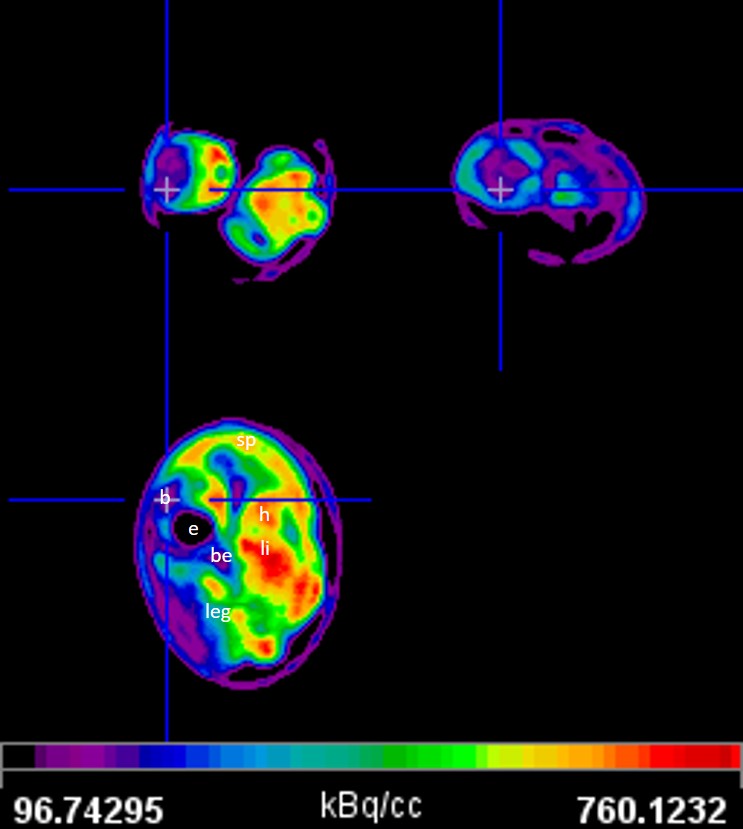


**Figure S4.**

Representative summed image of [^18^F]FET µPET of a chick embryo (EDD19), from 18-50 minutes post injection. The crosshair points at the brain (b).

*b*, brain; *sp*, spine; *e*, eye; *be*, beak; *h*, heart; *li*, liver

**References**

1. Stegmayr C, Stoffels G, Kops ER et al. (2019) Influence of Dexamethasone on O-(2-18F-Fluoroethyl)-L-Tyrosine Uptake in the Human Brain and Quantification of Tumor Uptake. Mol Imaging Biol; 21(1):168–74. DOI:10.1007/s11307-018-1221-z.

2. Stegmayr C, Bandelow U, Oliveira D et al. (2017) Influence of blood-brain barrier permeability on O-(2-18F-fluoroethyl)-L-tyrosine uptake in rat gliomas. Eur J Nucl Med Mol Imaging; 44(3):408–16. DOI:10.1007/s00259-016-3508-0.
